# Exposure to Hurricane-Related Flooding and Outcomes of Home Health Care Patients

**DOI:** 10.1001/jamahealthforum.2026.1758

**Published:** 2026-06-26

**Authors:** Arnab K. Ghosh, Mark A. Unruh, Hye-Young Jung, Hyunkyung Yun, Madeline R. Sterling

**Affiliations:** 1 Department of Medicine, Weill Cornell Medical College, Cornell University, New York, New York; 2 Department of Population Health Sciences, Weill Cornell Medical College, Cornell University, New York, New York; 3 Department of Health Services, Policy, and Practice, Brown School of Public Health, Providence, Rhode Island

## Abstract

This cohort study assesses the convergence of the rising threat of extreme weather events and increasing use of home health care through the lens of Hurricane Sandy.

## Introduction

Home health care (HHC) is a key tenet of the US health care system, with more than 3.3 million Medicare beneficiaries receiving these services, constituting $133.4 billion in expenditures.^1^ Older adults receiving HHC services are homebound, medically complex, frail, and often have functional and cognitive deficits, which make them particularly vulnerable to extreme weather events like hurricanes. Extreme weather events may inflict widespread disruption in an already decentralized system and network of HHC. For example, extreme weather events may prevent HHC agency nurses and other professionals from traveling to patients’ homes, interrupt communication systems, and halt delivery of supplies.^2^

Yet, the convergence of the rising threat of extreme weather events together with increasing HHC use^3^ has not been thoroughly investigated, despite prior work demonstrating vulnerabilities of HHC agencies and beneficiaries to disasters, including the COVID-19 pandemic.^4,5^ We used the setting of Hurricane Sandy, the third costliest storm in US history, to assess the association between exposure to hurricane-related flooding and outcomes of HHC recipients residing in New York City, New York; Connecticut; and New Jersey.

## Methods

The Weill Cornell Medicine Institutional Review Board deemed this cohort study exempt because the data were deidentified. Informed consent was waived because this was a secondary analysis of deidentified data. The Strengthening the Reporting of Observational Studies in Epidemiology (STROBE) reporting guideline was followed. We included beneficiaries who were admitted to HHC at the time of Hurricane Sandy’s impact (October 29, 2012), identified through claims data for a 20% national sample of Medicare fee-for-service beneficiaries continuously enrolled in Parts A/B, with functional assessment data captured by the Outcome and Assessment Information Set. Using US Geological Survey flood data, we defined the exposure group as patients residing in zip codes that experienced any flooding due to Hurricane Sandy, and the comparison group as HHC recipients in nonflooded zip codes within a 10-mile radius of flooded zip codes.

Outcomes specific to HHC were calculated and compared for the 2 groups: timely start of care, improved medication management, mean HHC length of stay, discharged to a skilled nursing facility, and discharged to the community. Race and ethnicity were self-reported and stratified into 3 groups for this study: Black, non-Hispanic White, and other (American Indian/Alaska Native, Asian/Pacific Islander, Hispanic, and other).

Continuous measures were compared using 2-sided *t* tests or Mann-Whitney tests where assumptions of normality were violated, and categorical measures were compared with χ^2^ tests. *P* values less than .05 indicate statistical significance. Analyses were conducted using SAS statistical software, version 8.3 (SAS Institute Inc) on April 13, 2026.

## Results

The sample included 761 beneficiaries (mean [SD] age, 77.3 [12.7] years; 499 female [66.6%] and 262 male [34.4%]; 115 Black [15.1%], 513 non-Hispanic White [67.4%], and 133 other [17.5%]); 432 were exposed to flooding, and 329 were not exposed (Table). Except by race and ethnicity and state of residence, there were no significant differences in demographic characteristics for HHC recipients in nonflooded vs flooded zip codes (mean [SD] age, 77.3 [12.1] vs 77.2 [13.1] years, respectively; *P* = .16; proportion female, 210 [63.8%] vs 289 [66.9%], respectively; *P* = .38) nor clinical characteristics (proportion with Alzheimer disease and related dementias, 119 [36.2%] vs 155 [35.9%], respectively; *P* = .93; congestive heart failure, 155 [50.5%] vs 229 [53.0%], respectively; *P* = .48).

**Table.** Characteristics and Home Health Care (HHC) Outcomes of Medicare Fee-for-Service Beneficiaries Exposed vs Not Exposed to Hurricane-Related Flooding

Characteristic	No. (%)		*P* value^a^
	Exposed to flooding^b^		
	No (n = 329)	Yes (n = 432)	
Age, mean (SD), y	77.3 (12.1)	77.2 (13.1)	.84
Sex			
Female	210 (63.8)	289 (66.9)	.38
Male	119 (36.2)	143 (33.1)	.38
Race and ethnicity			
Black	47 (14.3)	68 (15.7)	.58
Non-Hispanic White	237 (72.0)	276 (63.9)	.02
Other^h^	88 (20.4)	45 (13.7)	.02
Medicare eligibility			
Age	290 (88.2)	366 (84.7)	.17
Disability	37 (11.3)	61 (14.1)	.24
State of residence			
New York	214 (65.0)	214 (49.5)	<.001
New Jersey	91 (27.7)	129 (29.9)	.51
Connecticut	22 (6.7)	89 (20.6)	<.001
Select comorbidities and index			
Alzheimer disease and related dementias	119 (36.2)	155 (35.9)	.93
Congestive heart failure	166 (50.5)	229 (53)	.48
End-stage kidney failure	140 (42.6)	182 (42.1)	.91
Chronic obstructive pulmonary disease	92 (28)	136 (31.5)	.29
Diabetes	189 (57.5)	241 (55.8)	.65
Hip fracture	25 (7.6)	19 (4.4)	.06
Stroke	58 (17.6)	64 (14.8)	.35
Hierarchical condition category community score, mean (SD)	2.47 (1.8)	2.62 (1.9)	.40
HHC outcomes			
Timely start of care^c^	210 (63.8)	240 (55.5)	<.001
Improved medication management^d^	145 (44.1)	173 (40.1)	.08
Mean HHC length of stay, d^e^	70.6 (221.6)	83.5 (212.3)	.03
Skilled nursing facility admission at end of HHC episode^f^	53 (16.1)	72 (16.7)	.84
Discharged to community^g^	147 (44.7)	171 (39.6)	.04

^a^ Across all tests, violations of normality were tested.

^b^ Defined as residing in a zip code impacted by hurricane-related flooding.

^c^ Start of care defined as whether individual received HHC within 48 hours of referral, physician’s order (community-based), or after returning home (posthospitalization); analysis of this outcome variable was restricted to Medicare fee-for-service beneficiaries who were admitted to HHC 2 days before the hurricane’s landfall (October 27, 2012, and onwards) given the time-sensitive nature of the definition of timely start of care.

^d^ Defined as the home health episode during which patient had improved ability to take oral medication.

^e^ Total number of days HHC services received.

^f^ Whether HHC recipient was admitted to a skilled nursing facility.

^g^ Discharged to community defined as whether an individual was discharged to community after the home health episode.

^h^ Other includes American Indian/Alaska Native, Asian/Pacific Islander, Hispanic, and other.

A larger proportion of beneficiaries in nonflooded zip codes were successfully discharged to the community compared to those in flooded zip codes (147 [44.7%] vs 171 [39.6%], respectively; *P* = .04), in timely start of care (210 [63.8%] vs 240 [55.5%], respectively; *P* < .001), and a significant difference in mean (SD) HHC length of stay was observed (70.6 [221.6] days vs 83.5 [212.3] days, respectively; *P* = .03). No significant differences were found in discharge to a skilled nursing facility, or for improved medication management by flood zone status.

## Discussion

This cohort study found that HHC recipients impacted by flooding had lower rates of successful discharge to the community, timely start of care, and operationally meaningful longer HHC length of stay compared to HHC recipients not exposed to hurricane-related flooding. These findings underscore the vulnerability of HHC recipients to flooding and invite further questions about the incorporation of HHC into broader health care emergency planning efforts at local and regional levels.^2,6^ Our findings are subject to 3 key limitations, including unknown generalizability to the Medicare Advantage population, limited power to reveal differences in other health outcomes, and the inability to control for HHC agency characteristics because these data were not available in 2012. Future research should address these limitations and expand this work to other types of extreme weather events, differential impacts by extreme weather event type, geographical variation, and long-term health trajectories of HHC recipients.

## Notes

Supplement. Data Sharing Statement
