# Supplementary material for: Exposure to Hurricane-Related Flooding and Outcomes of Home Health Care Patients
Source: JAMA Health Forum. 2026 Jun 26;7(6):e261758. doi: 10.1001/jamahealthforum.2026.1758 (PMC13309862; doi:10.1001/jamahealthforum.2026.1758)
Supplement: Supplement. — Data Sharing Statement [file jamahealthforum-e261758-s001.pdf]

# Data Sharing Statement

Ghosh. Exposure to Hurricane-Related Flooding and Outcomes of Home Health Care Patients. *JAMA Health Forum*. Published June 26, 2026. doi:10.1001/jamahealthforum.2026.1758

**Data**
**Data available:** No

**Additional Information**
**Explanation for why data not available:** Data comes from CMS and requires a DUA
